# Control of Acute Dengue Virus Infection by Natural Killer Cells

**DOI:** 10.3389/fimmu.2014.00209

**Published:** 2014-05-13

**Authors:** Caroline Petitdemange, Nadia Wauquier, Juliana Rey, Baptiste Hervier, Eric Leroy, Vincent Vieillard

**Affiliations:** ^1^Centre d’Immunologie et des Maladies Infectieuses (CIMI-Paris), Université Pierre et Marie Curie, Sorbonne Universités, Paris, France; ^2^INSERM, U1135, Centre d’Immunologie et des Maladies Infectieuses (CIMI-Paris), Paris, France; ^3^Centre International de Recherches Médicales de Franceville, Franceville, Gabon; ^4^Metabiota Inc., San Francisco, CA, USA; ^5^CNRS ERL8255, Centre d’Immunologie et des Maladies Infectieuses (CIMI-Paris), Paris, France

**Keywords:** NK cells, dengue viral infection, cytokines, cytotoxicity, viral escape mechanisms

## Abstract

Dengue fever is the most important arthropod-borne viral disease worldwide, affecting 50–100 million individuals annually. The clinical picture associated with acute dengue virus (DENV) infections ranges from classical febrile illness to life-threatening disease. The innate immunity is the first line of defense in the control of viral replication. This review will examine the particular role of natural killer (NK) cells in DENV infection. Over recent years, our understanding of the interplay between NK cells and viral pathogenesis has improved significantly. NK cells express an array of inhibitory and activating receptors that enable them to detect infected targets while sparing normal cells, and to recruit adaptive immune cells. To date, the exact mechanism by which NK cells may contribute to the control of DENV infection remains elusive. Importantly, DENV has acquired mechanisms to evade NK cell responses, further underlining the relevance of these cells in pathophysiology. Hence, understanding how NK cells affect the outcome of DENV infection could benefit the management of this acute disease.

## Introduction

Dengue virus (DENV) is the most widespread arbovirus worldwide transmitted by mosquitoes of the Aedes genus and is responsible for major outbreaks leading to serious health and economical problems (1). Approximately 500,000 DENV cases progress to be a life-threatening disease each year causing up to 20,000 deaths (2). DENV is a member of the genus Flavivirus and is divided into four different serotypes (DENV1–DENV4). Early 1970, Halstead and Simasthien (3) suggested that primary infection with one serotype of DENV only confers short-term partial cross-protection against other serotypes. Furthermore, secondary heterologous infections contribute to the development of severe forms of dengue fever (DF) [dengue hemorrhagic fever (DHF) and dengue shock syndrome (DSS)] (4).

The clinical picture of primary DENV infection includes fever, headache, myalgia, arthralgia, and petechial rash. Patients rapidly develop high viremia for up to 6 days after the onset of fever. The rapid initiation of the hosts’ innate defense mechanisms might be a limiting factor in the development of DENV infection. In rare cases, patients may develop complications including plasma leakage and coagulation disorders, which may lead to a fatal outcome (5, 6). One of the most important questions in regards to dengue pathogenesis is the identity of the cells that play a crucial antiviral role during the innate immune response to DENV at the earliest stages of infection (7). Natural killer (NK) cells are a major component of the innate arm of the immune system. Although NK cells hold an early and central role early after number of viral infections, not only for viral containment but also for timely and efficient induction of adaptive responses, their role in the control of DENV infection is still poorly documented. The observation in the late 1970s that viral infections induce NK cell-mediated killing stimulated a frenzy of research focused on evaluating the role of these cells in defense against several viral infections and cancer. The clearest demonstration of this role derives from the growing number of cases of NK cell deficiency. Since the initial known case of a girl with multiple severe or disseminated herpesvirus infections, about 40 other unrelated cases have been described and were mainly associated with severe herpesvirus infections, and EBV- or HPV-related cancers (8). Major progress has been achieved since, in several important fields related to NK cells. NK cells represented a wonderful biological paradox in that they appeared fully competent to kill target cells and yet were clearly self-tolerant. As such, NK cells were “armed” but not dangerous. Scientists focused their attention on understanding how these potent killers were contained and controlled. We now appreciate that a precise balance of inhibitory and activating signals mainly regulates the functional outcome of these cells (9–11). A flood of information concerning multiple types of negative receptors on NK cells was gathered. Many of these receptors respond to stimulation by major histocompatibility complex (MHC) class-I molecules expressed on the surface of target cells and this is considered to be the predominant mechanism responsible for NK cell tolerance to self. These inhibitory NK receptors include ILT-2 and the CD94/NKG2A complex, which recognize HLA-G and HLA-E, respectively, whereas the inhibitory killer-cell immunoglobulin-like receptors (KIRs) recognize polymorphic MHC class-I molecules; in particular: KIR2DL1/KIR2DL2 and KIR2DL3 bind group 2 (C2) and group 1 (C1) HLA-C alleles, respectively, while KIR3DL1 recognizes HLA-Bw4 epitopes (12). Numerous studies have shown that these factors likely synergize to generate susceptibility or resistance to pathogens and disease (12, 13). In patients from southern Brazil, the susceptibility to DF is positively associated with the presence of KIR3DS1-Bw4, KIR3DL1-Bw4, KIR2DL1-C2, and KIR2DS1-C2 genes, and negatively associated with KIR2DL3-C1/C1 (14).

To destroy a target, NK cells also present several cell-surface activating receptors such as: NKG2D, DNAX accessory molecule-1 (DNAM-1), an adhesion molecule physically and functionally associated to Lymphocyte function-associated antigen 1 (LFA-1), and the natural cytotoxicity receptors (NCRs: NKp30, NKp44, and NKp46) (15, 16). Simultaneous interactions of certain of these activating receptors on NK cells with their specific ligands on the target cells, lead to the integration of different signals including the modulation of the cell-cycle, and together dictate the quality and intensity of the effector NK cell response (17). The relative contribution of each of the activating receptors to NK cytotoxicity against target cells differs, indicating the existence of an array of specific ligands that will be presented and their role in DENV infection further developed by Beltrán and Lopez-Verges, under the research topic “Protective Immune Response to Dengue Virus Infection and Vaccines: perspectives from the field to the bench.”

In this mini-review, we focused on what is currently known about the mounting of the NK cell response with its role in cytotoxicity and immunoregulation during DENV infection as well as the mechanisms acquired by the virus to evade NK cell killing.

## Activation of NK Cells after DENV Infection

Very early after infection, interferon-alpha (IFN-α) is the mainstay of host defenses. This type I, IFN, is a crucial mediator of the antiviral response directly inhibiting viral replication and modulating downstream immune responses to counteract viral spread (18). Elevated IFN-α plasmatic levels are observed shortly after onset of symptoms in children and adult DENV-infected patients (19, 20). Recently, Gandini et al. (21) have shown that DENV2 efficiently activated IFN-α production by plasmacytoid dendritic cells (pDCs), which produced up to 1000-fold more IFN-α than other cell types in response to virus exposure. The infected pDCs could then decrease DENV infection of monocytes. The importance of the IFN-α response is also illustrated by the increased lethality of IFN-α/β receptor knockout mice when administered DENV2 by intraperitoneal injection (22). Although it was shown that the *in vitro* pretreatment of cultured cells with IFN-α/β dramatically reduces DENV replication, type I IFN has little effect on DENV replication after viral replication has been established (23). Indeed, DENV can reach high titers (<10^9^ infectious doses per milliliter) in humans despite the induction of high levels of circulating IFN-α (19, 24). Therefore, it seems likely that DENV has evolved mechanisms to counteract the IFN response, a characteristic that is shared by many pathogenic viruses (25).

**Figure 1:**
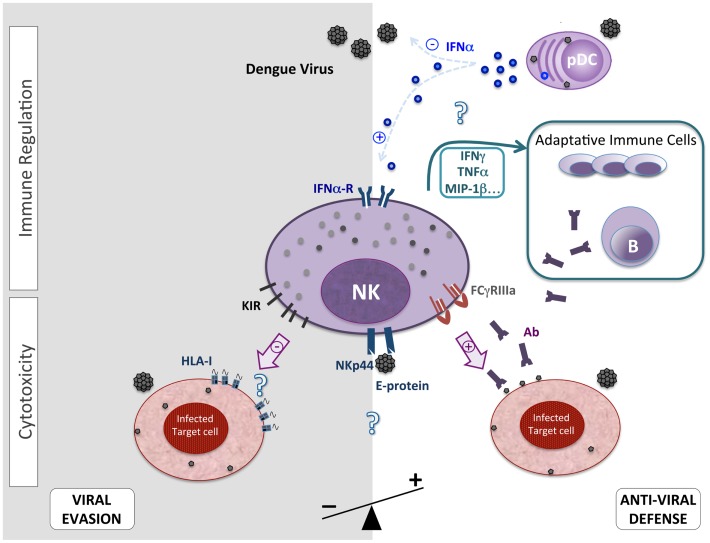
**Overview of the suggested NK cell features after acute and primary DENV infection**.

It has long been established that one of the main mechanisms accounting for the efficacy of the type I IFNs is their ability to activate NK functions (Figure 1); thus, they promote the accumulation and/or survival of proliferating NK cells by the STAT1-dependent induction of IL-15 secretion (26). This early activity of NK cells may be important for clearing primary DENV infection. In a sensitive mouse model, acute infection with DENV showed a rapid increase of NK cell levels (27). A significant increase in the frequency of NK cell circulation was also shown in patients who developed an acute DF (28). In addition, patients with a mild DF have elevated NK cell rates when compared to those with severe DF (29). Interestingly however, levels of circulating MIP-1β are higher in mild acute DF and are associated with higher NK cell frequencies (30). To characterize the primary NK response to DENV infection in mice, the phenotype of NK cells in the spleen was assessed by flow cytometric analysis. Three days after infection, the DENV-infected mice had twice as many NK cells than the mock-infected mice, and more than 50% of these NK cells expressed the early activation marker CD69, although only 5–10% of NK cells in the mock-infected animals expressed CD69 (27). Concomitantly, NK cells from DENV-infected patients display simultaneously high levels of CD69, HLA-DR, and CD38 (28, 29). For example, a significant increase in the percentage of CD69^+^-expressing NK cells was observed in DENV-infected patients at the early and acute phase of infection (days 1–5 with 29 vs. 13%), maintained at days 6–10 (24 vs. 18%), but decreased after 11 days (13 vs. 5%) (28). Altogether, these observations support the concept that DENV infection induces the selection and proliferation of a subset of activated NK cells further reinforcing their potentially important role during the early stages of the disease.

## NK Cell Function in the Protection Against DENV

### Cytokines produced by activated NK cells

Upon activation, NK cells may produce cytokines that favor the complete elimination of the disease and the infectious agent during the adaptive response, such as IFN-γ, TNF-α, G-CSF, and GM-CSF, as well as chemokines, such as MIP-1α, MIP-1β, and Rantes (31) (Figure 1). Numerous investigations have shown that DENV-infected patients presented significantly elevated levels of IFN-γ, G-CSF, and GM-CSF (20, 29, 32), whereas other reports suggest that TNF-α elevation could be associated with disease severity (33, 34). However, the results were obtained by measuring cytokine levels in sera collected from DENV-infected patients, and do not reveal which specific cells are activated and involved in their production. To date, the role played by NK cells in the production of these soluble factors is unknown. Deeper investigations will be necessary to precisely determine the implication of the immune-regulation by NK cells during the acute DENV infection.

### NK cell and their receptors during DENV infection

One of the most prominent functions of NK cells is the capacity to lyse virus-infected cells. When the balance of inhibitory/activating signals is in favor of activation, the engagement of activating receptors on the surface of NK cells leads to directed exocytosis of granules containing perforin and granzymes, which in turn elicit the disruption of the target cell membrane and/or the activation of apoptosis pathways within the infected cell (11). It has been shown that activated NK cells are cytotoxic against DENV-infected cells (35). Furthermore, a marker of cytotoxicity, the granule cytotoxic T cell intracellular antigen TIA-1, as well as two adhesion molecules, CD44 and CD11a, both involved in NK cell migration to various tissues and NK cytotoxicity, have been found to be significantly elevated on NK cells collected from acutely infected patients (28).

Hershkovitz et al. (36) have demonstrated the existence of a direct protein–protein interaction between recombinant DENV soluble envelope E protein and NKp44 (but not NKp30 or NKp46) that could be involved in the triggering of cytolysis (Figure 1). Using West Nile virus (WNV) like particles (VLPs) and WNV-infected cells, they have shown that E–NKp44 interaction triggers the secretion of cytotoxic granules contained in NK cells, the lysis of target cells, and the increased production of IFN-γ suggesting that flavivirus E proteins activate NK cytotoxic activity through NKp44 engagement.

Overall our understanding of the mechanisms involved in the cytolysis of target DENV-infected cells by NK cells is in its infancy. The receptors and signaling pathways essential to this major function are yet to be clearly identified. Recent investigations focusing on the various target ligands involved in the cytotoxic response by Beltrán and Lopez-Verges, under the Research topic “Protective Immune Response to Dengue Virus Infection and Vaccines: perspectives from the field to the bench” may bring some insight as to these questions.

### Activation of NK cells by ADCC responses during early DENV infection

Antibody-dependent cell-mediated cytotoxicity (ADCC) is another known mechanism by which NK cells recognize and lyse antibody-coated target cells, through the engagement of antibody binding to the Fc gamma receptor IIIA (CD16) (37). In an *in vitro* model, Kurane et al. (35) reported that human blood NK cells are cytotoxic against DENV-infected cells via direct cytolysis but also via ADCC (Figure 1). Indeed, PBMC collected from a DENV-infected patient successfully lysed DENV-infected cells that did not express MHC class-I molecules, and lysis of infected cells was significantly increased upon addition of anti-DENV1 and DENV2 monoclonal antibodies. The addition of sera from an individual without DENV antibodies did not lead to an increase in lysis of infected cells. More recently, García et al. (38) tested acute and convalescent patients’ sera for ADCC activity, differentiating mild and severe forms of DF. ADCC activity was observed with acute sera only in cases of DHF/DSS but not DF. However, using convalescent sera collected 1 year later, all samples induced ADCC activity. This suggests that the development of ADCC activity during the acute phase of the infection could be associated to the pathological manifestations of the severe syndrome and that the systematic development of ADCC activity after a primary infection, in convalescent patients could be associated with the development of severe forms during a subsequent heterologous DENV infection. However, other studies suggest a protective role for ADCC against DENV secondary infections. Indeed, higher ADCC activities have been associated with higher plasma neutralizing antibody levels and lower viral loads during secondary infection but solely if the patient was secondarily infected by DENV3 and not DENV2 (39).

Altogether, this data support a strong implication of ADCC after DENV infection, however, careful investigations are needed to determine its exact contribution both in the viral clearance during the initial acute phase of this infection and DENV pathogenesis in secondary infections.

## DENV Evasion of NK Cells

Co-existence of viruses and their infected hosts imposes an evolutionary pressure on both the virus and the host’s immune system. The host has developed an immune system able to attack viruses and virally infected cells, and viruses have developed an array of immune evasion mechanisms to escape being killed by the host’s immune system (40, 41). A wide variety of viruses have developed mechanisms to evade the NK cell response, and among these the flaviviruses are known to have developed particularly evolved mechanisms to escape NK cell-mediated lysis. Many viruses evade T-cells recognition by down-regulating MHC class-I restricted antigen presentation, whereas flaviviruses induce their cell-surface expression. This leaves infected cells less susceptible to NK lysing, MHC class-I molecules being able to effectively engage NK cell inhibitory receptors (11, 42, 43). Regarding DENV infection, a number of studies have demonstrated that DENV replicon expression is sufficient to enhance membrane expression of MHC class-I inducing a reduced susceptibility to NK lysis (42, 44). The way by which DENV induces MHC class-I up-regulation on the surface of infected cells has been associated with an activation of NF-κB, independently of IFN and without an increased synthesis of MHC class-I molecules (45). The exact mechanism(s) and especially the viral components involved are still obscure yet several hypotheses were proposed. The up-regulation of MHC class-I expression could be the result of: (i) an increase in the supply of peptides to the endoplasmic reticulum mediated by the transporter associated with antigen processing (TAP) (46) (ii) the expression of DENV non-structural protein(s) and/or viral RNA replication (44), and (iii) the accumulation of uncleaved C-prM protein during viral assembly (47).

The major biological consequence of DENV-induced MHC class-I increase is a profound reduction of NK cell lysis as shown in *in vitro* cytotoxicity assays (35, 44). Moreover, an aggregation of MHC class-I molecules at the surface of infected cells seems to lead to increased affinity with NK cell inhibitory receptors (44). Given that the kinetics of DENV viremia coincide with the peak of NK cell response, the modulation of MHC class-I expression may have physiological relevance in counteracting host NK cell defense. It is likely that there is a complex balance between DENV-induced activation of NK cells and subversion of NK killing by DENV-induced MHC class-I expression on infected cells. An additional component to take into account is the genetic background of the host since KIRs/HLA molecules combinations play a crucial role in the strength of NK cell inhibitory function (13), and this could clearly play important role on DENV infection susceptibility, protection, and severity.

These viral escape mechanisms certainly reflect evolutionary pressure exerted by the host immune response on the pathogen but also highlight the importance of NK cells in the defense against DENV infection.

## Conclusion

Overall, several lines of evidence converge to suggest that NK cells are activated early during an acute DENV infection, and produce major cytokines, such as IFN-γ, which participate in the control of the viral replication while promoting the development of an efficient adaptive immune response. In the future, it will be interesting to determine if and how DENV leaves an imprint on the NK receptor repertoire possibly favoring cells with a strong cytolytic potential, as previously shown, by us and other groups, with several other viruses, including chikungunya virus, which is yet an other arbovirus (48). Numerous studies have also shown that DENV increases MHC class-I and adhesion molecule expression, allowing the virus to escape NK cell lysis. Whilst underlining the importance of NK cells in DENV infection, these observations must be interpreted with great caution. Due to the lack of extensive phenotypic and functional studies in DENV-infected patients, we cannot conclude in favor of a beneficial or a deleterious role of the NK cells in the control and/or the evolution of the disease. Future studies based on the depletion of NK cells in a relevant animal model for DENV infection, will be able to unravel this fascinating topic.

## Conflict of Interest Statement

The authors declare that the research was conducted in the absence of any commercial or financial relationships that could be construed as a potential conflict of interest.
